# When is an herbivore not an herbivore? Detritivory facilitates herbivory in a freshwater system

**DOI:** 10.1002/ece3.4133

**Published:** 2018-05-07

**Authors:** Jessica L. Sanchez, Joel C. Trexler

**Affiliations:** ^1^ Department of Biological Sciences Florida International University Miami FL USA

**Keywords:** detritivory, diet evolution, diet quality, fatty acids, freshwater herbivore, herbivory, structural equation model

## Abstract

Herbivory is thought to be an inefficient diet, but it independently evolved from carnivorous ancestors in many metazoan groups, suggesting that plant‐eating is adaptive in some circumstances. In this study, we tested two hypotheses to explain the adaptive evolution of herbivory: (i) the Heterotroph Facilitation hypothesis (herbivory is adaptive because herbivores supplement their diets with heterotrophic microbes); and (ii) the Lipid Allocation hypothesis (herbivory is adaptive because algae, which have high lipid concentrations, are nutritionally similar to carnivory). We tested these hypotheses using enclosure cages placed in the Everglades and stocked with Sailfin Mollies (*Poecilia latipinna*), a native herbivore. Using shading and phosphorus addition (P), we manipulated the heterotrophic microbe and lipid composition of colonizing epiphyton and examined the effects of varying food quality on Sailfin Molly life history. Epiphyton grown in “shade only” conditions had a 55% increase in bacterial fatty acids and 34% lower ratios of saturated + monounsaturated to polyunsaturated fatty acids relative to the other treatments. Ratio of autotroph to heterotroph biovolume varied throughout the experiment, with a 697% increase at 3 weeks and 98% decrease at 6 weeks compared to the other treatments. Gut contents revealed that fish fed selectively on epiphyton to compensate for apparent deficiencies in the available food. Fish raised in “shade only” cages experienced the highest survival, which was best explained by autotrophic biovolume and algal‐ and bacterial‐derived fatty acids at 3 weeks (2–6× more likely than alternative models with ∆AICc > 2.00), and by percentage of bacterial fatty acids in the diet at 6 weeks (3–8× more likely than alternative models with ∆AICc > 2.00). There were no differences in fish growth among treatments. Autotrophic lipids play a role in early fish life history, but we did not find these to be the best predictors of life history later in the juvenile period. Instead, heterotrophic lipids facilitated the herbivorous diet and enhanced survival of juvenile fish in our experiment. Bacterial fatty acid content of the diet promoted herbivore survival, consistent with the Heterotroph Facilitation hypothesis. This is the first study to explicitly contrast Heterotrophic Facilitation and Lipid Allocation hypotheses for the adaptive evolution of herbivory in an aquatic system.

## 1 INTRODUCTION

Herbivory appears to be at an evolutionary disadvantage compared to omnivorous or carnivorous strategies (Sanchez & Trexler, 2016). Omnivores and carnivores consume animal prey that is high in nutritional value (Choat & Clements, 1998; Karban & Agrawal, 2002; Mattson, 1980; Sterner & Hessen, 1994), and omnivores have the additional advantage of supplementing their diets with abundant and easy to obtain plant items (Coll & Guershon, 2002; Diehl, 2003). Obtaining comparable energy from an exclusively herbivorous diet is difficult because food items are nutritionally variable and are usually accompanied by structural and/or biochemical barriers to assimilation (Chivers & Langer, 1994; Choat & Clements, 1998; Horn, 1989; Mattson, 1980; Porter & McDonough, 1984; Sterner & Hessen, 1994; and others). Furthermore, herbivores may be limited by foraging time and/or space by predators and competitors, by the ability to produce digestive or detoxifying enzymes (see Karban & Agrawal, 2002), or the amount of time it takes for food to pass through the gut (Bellwood, 1995; Bruggemann, Begeman, Bosma, Verburg, & Breeman, 1980; Choat & Clements, 1998; Horn, 1989). Despite these difficulties, there is evidence from many metazoan groups that herbivores evolved from carnivorous ancestors and that herbivory has been maintained alongside these animal‐containing diets in the majority of these lineages (e.g., Bellwood, 1976; Bellwood, Goatley, Brandl, & Bellwood, 1999; Espinoza, Wiens, & Tracy, 2004; Eubanks, Styrsky, & Denno, 2003; deMaintenon, 1999; Pauls, Graf, Haase, Lumbsch, & Waringer, 2008; Reisz & Frobisch, 2014; Van Damme, 1999; Vermeij, 1992; Vermeij & Lindberg, 2000).

Because few studies have addressed the adaptive significance of the herbivorous diet, we reviewed the freshwater herbivory literature to identify conditions where eating plants might be adaptive over eating animals (Sanchez & Trexler, 2016). We define freshwater “herbivory” as the consumption of algae and/or phytoplankton, and an “herbivore” as an organism that mainly eats these primary producers, but may indirectly consume detritus (consumes >50% primary producers). Furthermore, we define a “carnivore” as an organism that eats animals (consumes >50% animal material) and refer to an “omnivore” as an organism that eats both plants and animals (see Sanchez & Trexler, 2016 for a review). The term “food quality” is used to describe the nutritional worth of a diet item to a consumer and could be defined by macronutrient (e.g., nutritional ecology) or elemental (e.g., stoichiometry) composition, where food items are rich in protein or phosphorus, respectively. However, elements may not be ideal currencies to answer questions about organismal diets as they form the basis of the molecules that animals often select for (e.g., proteins, carbohydrates, and lipids; e.g., Sperfeld, Wagner, Halvorson, Malishev, & Raubenheimer, 2017), and thus, we use the stoichiometric definition of food quality with caution. Food quality may also be defined as the ratio of food energy content to that assimilated by consumers. Regardless of the convention used, “food quality” is a relative term and can only be interpreted relative to other diets (e.g., a diet item can be both high and low quality depending on the comparison diet), and respective of organismal diet adaptations (e.g., “high quality” is defined differently for carnivores vs. herbivores). Under these designations, we concluded that herbivory is favored when higher quality food is limiting, or when plants provide important dietary elements that are unavailable in carnivore diets, such as lipids (e.g., Martin‐Creuzburg, Beck, & Freese, 2011) or antioxidants (e.g., Pike, Blout, Bjerkeng, Lindstrom, & Metcalfe, 2007). Additionally, herbivores may overcome limiting resource quality by indirectly supplementing their diets with heterotrophic microbes that are associated with primary producers (see Sanchez & Trexler, 2016 for a review).

The idea that herbivores obtain nutrients from supplementary sources is well‐established (see White, 1985). In aquatic systems, herbivores (e.g., macroinvertebrates) are nutrient‐limited, and their nutrition is likely supported by detrital inputs (Hall, Likens, & Malcolm, 2001). The heterotrophic microbes that decompose detritus promote higher growth in macroinvertebrate families, compared to algal diets in both laboratory (e.g., Fuller & Fry, 1991; Fuller, Fry, & Roelofs, 1988; Fuller, Kennedy, & Nielsen, 2004) and field studies (e.g., Edwards & Meyer, 1990; Mulla & Lacey, 1976). Furthermore, growth rates of *Daphnia* spp. have been shown to increase when diets are supplemented with heterotrophic bacteria (e.g., Martin‐Creuzburg et al., 2011), emphasizing the importance of heterotrophs in the herbivorous diet. However, diets composed only of heterotrophic bacteria are of poor quality for herbivores (e.g., *Daphnia magna*), suggesting that they also rely on autotrophs for essential lipids like sterols or polyunsaturated fatty acids (e.g., Goulden, Henry, & Tessier, 1982; Martin‐Creuzburg, von Elert, & Hoffman, 2008; Martin‐Creuzburg, Wacker, & von Elert, 2005; Martin‐Creuzburg et al., 2011; Schmidt & Jonasdottir, 1997; Tessier, Henry, Goulden, & Durand, 1983; Weers & Gulati, 1997). The nutritional requirements of freshwater herbivores blur the distinction between herbivory and detritivory and emphasizes the idea that there are few “true” herbivores in nature (White, 1985).

Although previous studies have shown that aquatic herbivores rely heavily on nutrients originating from both heterotrophic microbes and autotrophic bacteria and algae (e.g., Belicka, Sokol, Hoch, Jaffe, & Trexler, 1993; Bowen, 1966; Martin‐Creuzburg et al., 2005, 2011; Smoot & Findlay, 2010), none have explicitly identified these dietary elements as facilitators of the evolution of herbivory. Here, we test two alternative hypotheses for the adaptive evolution of the herbivorous diet: (i) Heterotroph Facilitation hypothesis, which states that herbivory may be adaptive by supplementing herbivore diets with heterotrophic microbes (bacteria and/or fungi) that are indirectly consumed along with primary producers; and (ii) Lipid Allocation hypothesis, which states that consumption of autotrophic bacteria and algae, the primary source of essential fatty acids, may be as beneficial to individual life history as a carnivorous diet (Sanchez & Trexler, 2016). These hypotheses are not mutually exclusive, as the definition of heterotroph facilitation includes ingestion of autotrophic organisms. The key difference between these ideas lies in the nutritional source (heterotrophic vs. autotrophic microbes) that is the driver of life history.

**Figure 1:**
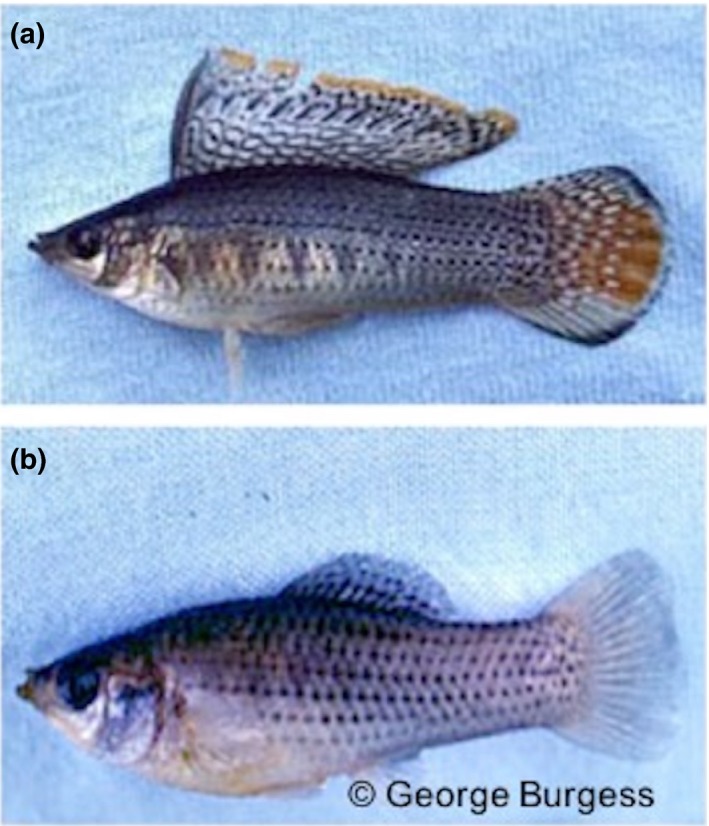
(a) Male Sailfin Molly (*Poecilia latipinna*). (b) Female Sailfin Molly (*Poecilia latipinna*). Images retrieved from the Florida Museum Ichthyology Collection, University of Florida, Gainesville, FL, © George Burgess

The Florida Everglades is an ideal system to test these adaptive hypotheses because periphyton mats are the primary basal resource in this area (Browder, Gleason, & Swift, 2010; Trexler, Gaiser, Kominoski, & Sanchez, 2015) and are composed of complex assemblages of autotrophs (green algae, diatoms, and cyanobacteria) and heterotrophs (fungi and bacteria; Gaiser et al., 2004). Both autotroph and heterotroph components of Everglades periphyton communities respond rapidly to changes in water chemistry (Gottlieb, Gaiser, & Lee, 2015; Noe, Childers, & Jones, 2001; Pan, Stevenson, Vaithiyanathan, Slate, & Richardson, 2000), such as when phosphorus is added, because the Everglades ecosystem is naturally oligotrophic (Gaiser et al., 2004). Furthermore, lipid profiles of Everglades primary and secondary consumers are comprised of both algal and bacterial‐specific fatty acids (Belicka et al., 1993), suggesting that both items are important in their diet. One of these species is the native Sailfin Molly (*Poecilia latipinna*), a small livebearing fish (Figure 1). Most *Poecilia* fishes are omnivorous (*P. vivipara*, Andrade, Nascimento, Gurel, & Medeiros, 2008; *P. mexicana*, Tobler, 2008), but stable isotope and gut content studies indicate that Sailfin Mollies are primarily herbivorous (Loftus, 2000, personal observation) and incorporate prokaryotic resources into their diet (Belicka et al., 1993). We used Sailfin Mollies held in enclosures in an Everglades marsh to test our alternative hypotheses of the adaptive advantage of the herbivorous diet. We predict that Sailfin Mollies will show increased growth and/or survival in response to increased dietary heterotrophic bacteria if the Heterotroph Facilitation hypothesis is the mechanism supporting the evolution of herbivory in the Everglades. Alternatively, Sailfin Mollies will show increased growth and/or survival in response to algal‐derived fatty acids if the Lipid Allocation hypothesis is supported by our study.

## 2 METHODS

We maintained juvenile Sailfin Mollies in cages in the Everglades from September 17 to October 29, 2015, to evaluate the effects of varying herbivorous diets on fish growth and survival. The 24 cages were 1‐m^2^ and had five surfaces covered in 1‐mm mesh (sides and bottom) and were open at the top. The cages were randomly placed in a slough located in the central Everglades (25°49′41.23″N, 80°37′53.41″W), with an average depth of 30 cm and temperature of 29.4 ± 1.2°C. Light and temperature were tracked throughout the experiment using HOBO^®^ data loggers. Artificial vegetation strips (2.54 cm wide) made of black plastic sheeting (0.154 mm thick) attached to wire frames for a total of 150 strips per frame, simulating natural stem density of this area (described in Chick et al., 2008), were added to each cage. The length of the strips was trimmed to water depth (approximately 28 cm) in the field so that they did not float on the surface and shade the water column. Periphyton was collected from the slough, cleaned of invertebrates, and 2,000 ml was placed into each cage to encourage growth of epiphytic algae on the artificial vegetation strips. An initial periphyton sample was brought back to the laboratory on ice and subsequently frozen for nutrient and lipid analyses (ambient periphyton). Sailfin Mollies were born in the laboratory and raised on Tetramin^®^ flake food for 6 weeks prior to the start of the experiment. They were measured (average standard length, SL) and transplanted to the field cages (*n* = 6 fish per cage; *N* = 36 total fish/treatment) 1 week following cage setup. This lag‐time allowed epiphyton to colonize the artificial vegetation strips prior to the addition of consumers. For detailed experimental setup, refer to Figures S1–S2 located in the supplementary material.

We manipulated colonizing epiphyton by adding phosphorus (P) and manipulating light (shade or light) to create a gradient of food quality for herbivores. Because the Everglades is a naturally oligotrophic system, both autotrophic and heterotrophic species within Everglades periphyton mats can be easily manipulated by addition of phosphorus. Each cage was randomly assigned to one of four treatments: (i) light + P; (ii) light only; (iii) shade + P; (iv) or shade only. Phosphorus (Na_2_HPO_4_) was added at a concentration of 15 μg/L weekly to “shade + P” and “light + P” cages. Previous studies manipulated the concentration of P across the Everglades landscape to understand the resulting changes to basal resources (e.g., Gaiser et al., 2005; McCormick & O'Dell, 1996; McCormick, Rawlik, Lurding, Smith, & Sklar, 1996; Noe et al., 2001). They found that low and intermediate P concentrations induced changes in Everglades primary producers, but high concentrations resulted in a phase shift (e.g., Gaiser et al., 2005). The lower and intermediate nutrient concentrations occur in nature, in areas where Sailfin Mollies are native. Therefore, we chose the intermediate concentration (15 μg/L) in order to manipulate epiphyton composition within the natural dietary range of Sailfin Mollies. Following dosing, these cages were wrapped with 3‐mm clear plastic to prevent P from seeping and potentially affecting nearby cages. Everglades periphyton incorporates P very quickly (Noe, Scinto, Taylor, Childers, & Jones, 2003); therefore, plastic covers were removed after 24 hr to permit water circulation. Shading was accomplished by covering cages with three sheets of glasshouse shade cloth to achieve approximately 75% reduction in ambient light (modified methods of Fuller et al., 2004).

Epiphyton, periphyton, and biofilms growing on the mesh cages were all potential herbivorous diet items available to grazing by fish. At 3 and 6 weeks, a sample of periphyton, a 5 × 5 cm scrape taken from the mesh wall inside the cage (herein referred to as “biofilm”), and 30 plastic strips were removed from each cage and brought back to the laboratory. At 3 weeks, two fish from each cage were euthanized with an overdose of MS‐222, and the remaining fish were returned to their respective cage. At 6 weeks, all remaining fish were measured, euthanized, and brought back to the laboratory on ice. Fish lacking gonopodial development (gonopodium, the male sexual organ) were dissected to assess fecundity.

Potential food items were processed for molecular analyses in the laboratory. Because plastic strips were various lengths from field trimming, standardized 30.5 cm sections from each were scraped of epiphytic algae. Subsamples of epiphyton, periphyton, and biofilm scrapes were kept for heterotroph and autotroph abundance estimates. Known volumes of epiphyton, periphyton, or biofilms were stained with either DAPI (4′,6‐diamidino‐2‐phenylindole) for bacteria (Hobbie, Daley, & Jasper, 1977), or labeled lectin (fluorescien‐labeled wheat germ agglutinin) for fungal counts (e.g., Wanchoo, Lewis, & Keyhani, 2009). Heterotrophs were counted under a microscope at 40× using epifluorescence, and autotrophs were counted using standard light microscopy at 40× magnification. Counts were transformed into total cells/ml of material. Volume of bacteria, fungi, and common algal species was estimated by taking measurements from 20 to 30 representative organisms for each from high‐definition photos and multiplied by total cells/ml to yield biovolume (μm^3^/ml) estimates.

The remaining samples (including fish) were freeze‐dried and prepped for fatty acid (sent to Microbial ID laboratory, Newark, DE) and stoichiometric analyses (CNP; sent to Southeastern Research Center, Florida International University, Miami, FL). Elements (CNP) are likely not ideal currencies for nutrition, but we measured the ratio of carbon to phosphorus, C:P, and ratio of nitrogen to phosphorus, N:P (molar ratios) to compare nutritional and stoichiometric methodologies. Fatty acid data were categorized by diet tracers (Table 1; Belicka et al., 1993) and further organized into polyunsaturated fatty acids (PUFAs), saturated fatty acids (SAFAs), and monounsaturated fatty acids (MUFAs). Fatty acids were also organized by common essential fatty acids that are known to affect fish growth and development: eicosapentaenoic acid (EPA), docosahexaenoic acid (DHA), and arachidonic (ARA) (see Saikia & Nandi, 2010 for a review). In addition to fatty acid and nutrient analyses, algal, bacterial, and fungal biovolume were used to calculate a ratio of autotrophic to heterotrophic organisms (A:H biovolume ratio). These metrics were analyzed in fish tissues and potential food sources to evaluate their influence on fish life history.

**Table 1:** Sources of fatty acid tracers used in this study (modified from Belicka et al., 2012)

Carbon Source (grouped by fatty acids used in this study)	References
Bacteria (15:0i, 15:0a, 15:0n, 17:0i, 17:0a, 17:0n, 18:1w7, 19:1)	
Odd carbon number fatty acids, 15:0i, 15:0a, 17:0i, 17:0a, 18:1w7	Findlay and Dobbs (1993), Napolitano (1999) and references therein, Volkman et al. (1980)
Algae (16:3, 18:3w3, 18:4, 18:3w6, 20:4w6, 20:5w3 (EPA), 20:4, 22:4w6, 22:5w3, 22:5w6, 22:6w3)	
14:0, 16:1w7: multiple sources, but high in diatoms and some cyanobacteria	Napolitano (1999) and references therein
C_16_ PUFA: green algae and diatoms	Kates and Volcani (1966), Cranwell et al. (1990), Napolitano (1999)
18:3w3: green algae, cyanobacteria	Ahlgren et al. (1992), Dalsgaard et al. (2003)
18:3w6: cyanobacteria	Napolitano (1999)
18:4w3, 18:5w3, 22:6w3: dinoflagellates	Ahlgren et al. (1992), Dalsgaard et al. (2003)
20:5w3, ratio of 20:5w3 to 22:6w3: diatoms	Napolitano (1999), Dalsgaard et al. (2003)

### 2.1 Statistical analyses

Growth curves of poeciliid fishes are more strongly asymptotic in males than females (Snelson, 1989), a phenomenon well‐described for Sailfin Mollies (Snelson, 1982; Travis, Farr, McManus, & Trexler, 1989). There were a few mature males at the end of the experiment; however, there were no developing embryos found in the ovaries of the females, so growth curves were treated as if fish had not yet matured. Fish standard length (mm) measurements at 0, 3, and 6 weeks were analyzed using two‐way analysis of variance (ANOVA). Fish standard length (mm) measurements by week were analyzed using the quadratic equation. Growth rates were estimated by dividing the slope at 2/3 of that curve by the number of days to obtain the growth of Sailfin Mollies per day in mm (following Trexler & Travis, 1990). A logit model with maximum likelihood was fit to fish survival data to predict the probability of survival, *p*, where logit(*p*) = log (*p*/1 − *p*). Temperature and light availability, potential influences on fish growth and survival, were analyzed for each treatment using one‐way ANOVA.

Multiple potential diet items were present in the experimental cages (biofilm, epiphyton, and periphyton described above); therefore, it was important to determine which diet items had the strongest influence on fish size and survival. We assumed that items that best predict fish life history were those that dominated the diets of fish in the experimental cages. Several food‐quality variables were measured for all potential diet types: ratio of carbon to phosphorus (C:P), ratio of nitrogen to phosphorus (N:P), relative fatty acid content, percentage of algal‐ and bacterial‐derived fatty acids, fatty acid class (PUFA, SAFA, MUFA, ratio of SAFA + MUFA: PUFA), essential fatty acids (EPA, DHA, ARA, ratio of EPA:DHA), A:H biovolume, and proportion of edible algae (proportion of green algae relative to cyanobacteria). Stoichiometry of algal types (C:P) was analyzed using one‐way Analysis of Variance (ANOVA) and Tukey post hoc tests. Algal species from epiphyton, periphyton, and fish guts were analyzed using two‐way multivariate analysis of variance (MANOVA) with Tukey post hoc tests. To determine the probability that a fish would eat a diet item based on its availability in the environment, we calculated Ivlev's Electivity Index, *E*_*i*_ = (*r*_*i*_ − *p*_*i*_)/(*r*_*i*_ + *p*_*i*_), where *r*_*i*_ = the proportion of the item found in the gut and *p*_*i*_ = the proportion of the item found in the environment (Ivlev 1961). Calculated indices were rounded to the nearest whole number. A value of *E*_*i*_ < 0 suggests that fish are avoiding the dietary item, *E*_*i*_ > 0 suggests that the fish are actively selecting the item, and *E*_*i*_ = 0 means that items are eaten in proportion to their availability in the environment. These were calculated for each treatment at both 3 and 6 weeks.

Relative fatty acid content of all samples was calculated by dividing the mass spectrometry peak area for each by the mg of dry weight of each sample. Although not a quantitative measure, it allowed us to compare relative fatty acid content across experimental treatments. These relative values were analyzed using two‐way ANOVA with post hoc tests. Two‐way ANOVAs were also used to assess any differences in the percentage of algal and bacterial‐derived fatty acids across treatments. Fatty acid classes (PUFA, SAFA, MUFA) and essential fatty acids (EPA, DHA, ARA) comprising each algal type were analyzed using MANOVA tests, followed by Tukey multivariate comparison tests (ln transformed). Ratios of fatty acid classes (SAFA + MUFA: PUFA, ln transformed) and essential fatty acids (EPA:DHA, log + 1 transformed) were analyzed using two‐way ANOVA. Biovolume of heterotrophs and autotrophs were converted to ratios (A:H biovolume), natural log‐transformed (ln), and analyzed using two‐way ANOVA. Proportion of edible algal species comprising each of the diet type was analyzed using two‐way ANOVA.

Epiphyton and biofilm were not statistically different from each other across all measured variables, so biofilm was dropped from future analyses. Of the 14 measured characteristics, variables that were statistically different (α ≤ 0.05) between epiphyton and periphyton were used as independent variables in Discriminant Function Analysis, with diet type as the grouping variable. These were C:P, A:H biovolume, SAFA + MUFA:PUFA, EPA:DHA, and percent of bacterial fatty acids. Discriminant scores for the function explaining the most variance were used as input variables for Structural Equation Models (SEM; Grace, 2006), which were fit using AMOS (Arbuckle, 2003). Using Principal Component Analysis, fish size and survival rates were collapsed into a single score that was also an input for SEMs.

We used SEMs to evaluate the information in alternative hypothesized pathways that our treatments (light and nutrient manipulation) may affect the consumers through their impact on primary producers. The first set of three models was designed to test the linkages between potential food items and fish life history. Paths were varied between epiphyton, periphyton, and fish life history in each model. Models were compared using Akaike's Information Criterion (AIC) by calculating ∆AICc (∆AICc = AIC_*i*_ − min AICc, where *i* = model *i*), Akaike weight (${\text{AIC}}_{w}=\left(e^{\left(-0.5*\Delta {\mathrm{AIC}}_{i}\right)}\right)/\Sigma \left(e^{\left(-0.5*\Delta {\mathrm{AIC}}_{r}\right)}\right)$, Relative likelihood (*L*_r_), and Evidence Ratios (*w*_min_/*w*_j_, where *w*_min_ = AIC_w_ for the model with the smallest ∆AICc and *w*_*j*_ **= AIC_w_ for the current model; Anderson & Burnham, 1990). Path coefficients (regression weights) were assessed to determine which variables best‐predicted life history. Following Anderson and Burnham (1990), models with ∆AICc <2.0 were considered equally explanatory. These models were fit for both 3 and 6 weeks.

We tested the alternative adaptive hypotheses by determining which food quality parameter influenced fish life history. The Heterotroph Facilitation hypothesis predicts that heterotrophs in the diet promote herbivore life history, and the Lipid Allocation hypothesis predicts that algal‐derived fatty acids are driving herbivore success. Therefore, we chose to evaluate A:H biovolume (measure of heterotroph and autotroph abundance), percentage of bacterial fatty acids (measure of bacterial quality), and SAFA + MUFA:PUFA ratios (algal‐derived fatty acids; measure of algal quality) as independent variables in a second set of SEMs designed to test the adaptive hypotheses. Paths were varied between these three diet variables and fish life history to produce a total of seven models. Similar to the first SEMs, models were compared using AIC.

## 3 RESULTS

### 3.1 Epiphyton

#### 3.1.1 3 weeks

The cages differed in phosphorus availability, but this did not translate to differences in epiphyton stoichiometry at 3 weeks. Ratios of C:P and N:P were similar for epiphyton grown in all treatments (*F*_3,8_ = 0.079, *p* = .970 and *F*_3,8_ = 0.367, *p* = .779, respectively).

Unlike stoichiometry, autotroph species composition was affected by light. Epiphyton samples collected at 3 weeks were comprised of similar algal species among treatments (Wilks’ Lambda = 0.053, *F*_15,11_ = 0.912, *p* = .588), but differed in relative abundance of edible algal types. Specifically, light drove the proportion of edible algae comprising epiphyton (light: *F*_1,8_ = 11.487, *p* = .010), where epiphyton from the “light only” treatments had 18% higher relative abundance of diatoms, solitary green, and filamentous green species than “light + P” epiphyton, and 94% higher abundance of these species than the shaded treatments. Furthermore, the shaded treatments were comprised of 50% inedible species (filamentous and coccoid cyanobacteria), as compared to 3% and 18% for “light only” and “light + P,” respectively.

Biovolume differed between light and shade treatments. The biovolume of heterotrophs (*F*_3,8_ = 0.415, *p* = .747) were not different between treatments; however, “shade + P” and “shade only” epiphyton was comprised of 238% and 887% greater autotrophs (respectively) compared to the other treatments (Light: *F*_1,8_ = 5.430, *p* = .048; P: *F*_1,8_ = 5.913, *p* = .041). Consequently, the ratios of A:H biovolume for “shade + P” and “shade only” epiphyton were approximately 140% and 697% greater than the light treatments, respectively (Light: *F*_1,8_ = 8.820, *p* = .018).

The relative abundance of types of fatty acids was affected by the both light and P treatments. There were no differences in the relative fatty acid content of epiphyton (*F*_3,8_ = 1.348, *p* = .279), the percentage of algal‐derived fatty acids (*F*_3,8_ = 1.534, *p* = .279) or the percentage of bacterial‐derived fatty acids (*F*_3,8_ = 0.299, *p* = .825). The relative abundances of PUFA's and SAFA's comprising the 3‐week epiphyton samples were driven by both light and nutrient addition (Wilks’ Lambda = 0.162, *F*_9,15_ = 10.31, *p* = .009), where “light only” epiphyton had approximately 59% higher PUFAs and “light + P” epiphyton had 8% higher SAFAs than the other treatments. However, only light drove the relative abundance of MUFAs (Wilks’ Lambda = 0.135, *F*_3,15_ = 12.811, *p* = .005). The shaded treatments had 10% higher MUFAs than the light treatments. Nutrient addition affected the SAFA + MUFA:PUFA ratios, where “light + P” and “shade + P” epiphyton had approximately 61% and 27% higher ratios relative to the other epiphyton types, respectively (phosphorus: *F*_1,8_ = 28.946, *p* = .002). Epiphyton grown in different treatments were not significantly different in EPA, DHA, and ARA (Wilks’ Lambda = 0.288, *F*_9,15_ = 0.915, *p* = .543). For a summary of results, refer to Table 2.

**Table 2:** Summary of results showing differences between experimental treatments for epiphyton, periphyton and fish tissues at 3 weeks

Metric	Epiphyton				Periphyton				Fish tissues			
	Light + P	Light only	Shade + P	Shade only	Light + P	Light only	Shade + P	Shade only	Light + P	Light only	Shade + P	Shade only
C:P	ns	ns	ns	ns	ns	ns	ns	ns	▲	▲	▼	▼
N:P	ns	ns	ns	ns	ns	ns	ns	ns	ns	ns	ns	ns
A:H biovolume	▼	▼	▲	▲	▲	▼	▲	▼	—	—	—	—
Relative FA content	ns	ns	ns	ns	ns	ns	ns	ns	ns	ns	ns	ns
Percent algal FA (%/wt)	ns	ns	ns	ns	ns	ns	ns	ns	ns	ns	ns	ns
Percent bacterial FA (%/wt)	ns	ns	ns	ns	ns	ns	ns	ns	ns	ns	ns	ns
FA ratio	▲	▼	▲	▼	ns	ns	ns	ns	ns	ns	ns	ns
EPA:DHA	ns	ns	ns	ns	ns	ns	ns	ns	ns	ns	ns	ns
ARA (%/wt)	ns	ns	ns	ns	ns	ns	ns	ns	ns	ns	ns	ns
Edible algal spp.	▲	▲	▼	▼	▲	▲	▼	▼	ns	ns	ns	ns

FA ratio = SAFA + MUFA:PUFA ratio. Upward facing triangles indicate relatively high values, whereas downward facing triangles indicate relatively low values. Values that are not statistically significant are indicated by “ns”. Blanks indicate metrics that could not be measured.

#### 3.1.2 6 weeks

Stoichiometric differences between treatments were revealed at 6 weeks. The C:P ratio of epiphyton was influenced by nutrient addition (phosphorus: *F*_1,8_ = 5.316, *p* = .05), where epiphyton grown in “light + P” and “shade + P” cages had 28% and 3% lower C:P ratios than the other treatments. However, there were no differences in N:P ratios of epiphyton growing in the different treatments (*F*_3,8_ = 2.703, *p* = .116).

Differences in autotroph species composition disappeared at 6 weeks. There were no differences in algal community structure (Wilks’ Lambda = 0.004, *F*_15,3_ = 1.407, *p* = .433) or in edible algae proportions across treatments (*F*_3,8_ = 1.125, *p* = .395).

Biovolume of autotrophs and heterotrophs was affected by both light and P at 6 weeks. Shaded treatments showed an 85% and 75% (“shade + P” and “shade only,” respectively) decrease in heterotroph biovolume relative to light treatments (*F*_3,8_ = 1.570, *p* = 0.271). Conversely, “light + P” treatments showed 65% decreased autotrophic biovolume (Light × P: *F*_1,8_ = 36.72, *p* < .0001) relative to all other treatments. As a result, light treatments had relatively low A:H ratios (approx. 98% decrease) compared to shaded treatments (Light: *F*_1,8_ = 5.088, *p* = .04). These ratios also increased in magnitude from 3‐week epiphyton.

Similar to 3‐week epiphyton, the relative abundance of types of fatty acids was affected by the both light and P treatments. There were no differences in the relative fatty acid content of 6‐week epiphyton (*F*_3,8_ = 0.254, *p* = .857) or the percentage of algal fatty acids (*F*_3,8_ = 1.580, *p* = .269). Differences in bacterial fatty acid composition became evident at 6 weeks (Light: *F*_1,8_ = 8.854, *p* = .018), where the “shade only” and “shade + P” treatments had 55% and 28% higher percentages than the other treatments, respectively. The relative abundance of PUFA's, SAFA's and MUFA's, and the SAFA + MUFA:PUFA ratios were the same (Wilks’ Lambda = 0.415, *F*_9,15_ = 0.713, *p* = .690 and *F*_15,3_ = 0.075, *p* = .591, respectively). In addition, epiphyton grown in different treatments were not significantly different in EPA, DHA, and ARA (Wilks’ Lambda = 0.234, *F*_9,15_ = 1.337, *p* = .299). For a summary of results, refer to Table 3. For detailed epiphyton results for both 3‐ and 6‐week time periods, refer to Table S1 located in the supplementary material.

**Table 3:** Summary of results showing differences between experimental treatments for epiphyton, periphyton and fish tissues at 6 weeks

Metric	Epiphyton				Periphyton				Fish tissues			
	Light + P	Light only	Shade + P	Shade only	Light + P	Light only	Shade + P	Shade only	Light + P	Light only	Shade + P	Shade only
C:P	▼	▲	▼	▲	ns	ns	ns	ns	▲	▲	▼	▼
N:P	ns	ns	ns	ns	ns	ns	ns	ns	▲	▲	▼	▼
A:H biovolume	▼	▼	▲	▲	▼	▲	▼	▼	—	—	—	—
Relative FA content	ns	ns	ns	ns	ns	ns	ns	ns	▲	▲	▼	▼
Percent algal FA (%/wt)	ns	ns	ns	ns	ns	ns	ns	ns	ns	ns	ns	ns
Percent bacterial FA (%/wt)	▼	▼	▲	▲	ns	ns	ns	ns	ns	ns	ns	ns
FA ratio	ns	ns	ns	ns	ns	ns	ns	ns	ns	ns	ns	ns
EPA:DHA	ns	ns	ns	ns	ns	ns	ns	ns	▼	▼	▼	▲
ARA (%/wt)	ns	ns	ns	ns	ns	ns	ns	ns	▼	▼	▼	▲
Edible algal spp.	ns	ns	ns	ns	ns	ns	ns	ns	▲	▲	▼	▼

FA ratio = SAFA + MUFA:PUFA ratio. Upward facing triangles indicate relatively high values, whereas downward facing triangles indicate relatively low values. Values that are not statistically significant are indicated by “ns”. Blanks indicate metrics that could not be measured.

### 3.2 Periphyton

#### 3.2.1 3 weeks

At 3 weeks, stoichiometric ratios of periphyton were consistent across treatments. Ratios of C:P and N:P were not different across treatments or from ambient periphyton (*F*_3,8_ = 0.551, *p* = .662 and *F*_3,8_ = 0.231, *p* = .872, respectively). Periphyton C:P and N:P was different from that of epiphyton (*F*_1,8_ = 142.32, *p* < .001 and *F*_1,8_ = 19.83, *p* < .001, respectively), as periphyton had 110% higher C:P and 23% higher N:P ratios.

Autotroph species composition of 3‐week periphyton was driven by light. Periphyton samples were similar in algal composition among treatments (Wilks’ Lambda = 0.514, *F*_6,14_ = 0.920, *p* = .509), but differed in relative abundance of edible algal types. Light drove the proportion of edible algae comprising periphyton (Light: *F*_1,8_ = 5.23, *p* = .05), where the light treatments had approximately 63% higher abundance of edible species than the shaded treatments.

The A:H biovolume ratios of 3‐week periphyton were driven by P‐addition. Periphyton grown in the “light + P” and “shade + P” treatments had 24% and 425% higher A:H biovolume than “light only” and “shade only” periphyton, respectively (phosphorus: *F*_1,8_ = 0.129, *p* = .003).

The relative abundance of types of fatty acids in periphyton was similar across treatments at 3 weeks. The percentage of algal and bacterial‐derived fatty acids (Wilks’ Lambda = 0.743, *F*_6,14_ = 0.411, *p* = .884), and the relative fatty acid content (*F*_3,8_ = 0.919, *p* = .474) were not different among treatments.

The proportion of PUFAs, SAFAs, and MUFAs (Wilks’ Lambda = 0.452, *F*_9,15_ = 0.633, *p* = .752) as well as the SAFA + MUFA:PUFA ratios of 3‐week periphyton were similar across treatments (*F*_3,8_ = 1.392, *p* = .314). Essential fatty acid composition (EPA, DHA, ARA) of periphyton was not different across treatments (Wilks’ Lambda = 0.635, *F*_6,14_ = 0.595, *p* = .730), but periphyton had nondetectable levels of DHA (i.e., 0.0% by weight), which was significantly lower than epiphyton (*F*_1,8_ = 88.17, *p* < .0001). For a summary of results, refer to Table 2.

#### 3.2.2 6 weeks

Similar to 3‐week periphyton, stoichiometric ratios of periphyton were not different across treatments. Ratios of C:P and N:P were consistent across treatments (*F*_3,8_ = 0.487, *p* = .701 and *F*_3,8_ = 0.438, *p* = .732, respectively); however, ambient periphyton was stoichiometrically different than 6‐week periphyton from the experimental treatments (*F*_4,9_ = 5.965, *p* = .013), with 64% and 2% greater C:P and N:P ratios, respectively.

Autotroph species composition of 6‐week periphyton was not driven by light, in contrast to periphyton at 3 weeks. Periphyton samples collected at 6 weeks were similar in algal species composition among treatments (Wilks’ Lambda = 0.191, *F*_15,3_ = 0.980, *p* = .510) and in the proportion of edible algal species (*F*_3,8_ = 0.757, *p* = .549).

The A:H biovolume ratios of 6‐week periphyton were driven by light and nutrients. Periphyton in the “light + P” cages had 96% lower A:H ratio than “light only” periphyton, and 74% lower ratio than the shaded treatments (Light × P: *F*_1,8_ = 5.211, *p* = .05).

The relative abundance of types of fatty acids in periphyton were similar across treatments at 6 weeks. The percentage of algal and bacterial‐derived fatty acids (Wilks’ Lambda = 0.679, *F*_6,14_ = 0.499, *p* = .799) and the relative fatty acid content (*F*_3,8_ = 0.170, *p* = .913) were not different among treatments. The proportion of PUFAs, SAFAs, and MUFAs (Wilks’ Lambda = 0.453, *F*_9,15_ = 0.630, *p* = .755) as well as the SAFA + MUFA:PUFA ratios were similar across treatments (*F*_3,8_ = 0.961, *p* = .457). Essential fatty acid composition (EPA, DHA, ARA) of 6‐week periphyton was not different across treatments (Wilks’ Lambda = 0.703, *F*_3,15_ = 1.125, *p* = .395), but periphyton was significantly lower in DHA than epiphyton (*F*_1,8_ = 50.01, *p* < .001). For a summary of results, refer to Table 3. For detailed periphyton results for both 3‐ and 6‐week time periods, refer to Table S2 located in the supplementary material.

### 3.3 Fish

#### 3.3.1 3 weeks

**Figure 2:**
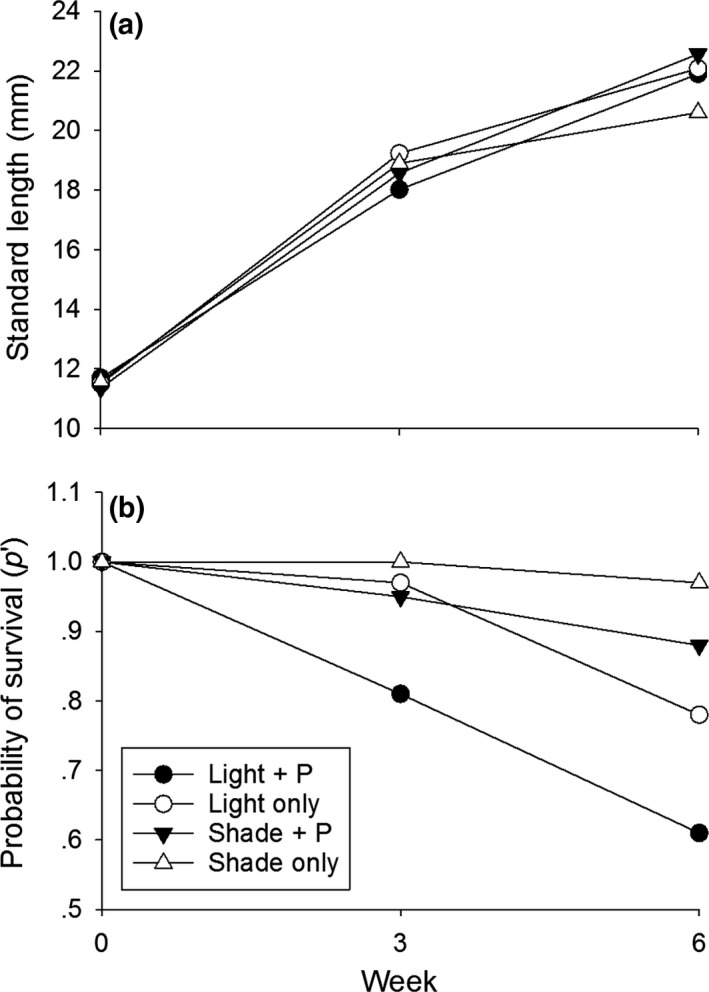
(a) Standard length (mm) of juvenile Sailfin Mollies raised on biofilms grown in various treatments. (b) Probability of survival (p′) of juvenile Sailfin Mollies showing high survival of those grown in “shade only” treatments

Juvenile Sailfin Molly survival, but not growth rate, was affected by the treatments. There were no differences in the sizes of juvenile fish stocked in each cage at the start of the experiment (*F*_3,8_ = 0.207, *p* = .891). The light cages were approximately 2°C warmer than the shaded cages (*F*_3,51_ = 7.617, *p* < .0001), but this did not translate into differences in fish growth, as all fish were similar sizes at week 3 (*F*_3,8_ = 1.597, *p* = .265). However, there were differences in fish survival among treatments. Specifically, fish in the “shade only” had the greatest survival compared to all other treatments (Χ^2^ = 14.979, *p* = .001). Fish reared in the “light + P” treatment experienced the lowest survival, which was 30% less than fish in the “shade only” treatment (Figure 2b).

Stoichiometric differences in fish tissues were evident at 3 weeks. Fish reared in the experimental treatments had 81% greater C:P ratios and 73% greater N:P ratios in their tissues relative to initial, laboratory‐reared fish‐fed commercial food (C:P, *F*_4,9_ = 5.293, *p* = .018; N:P, *F*_4,9_ = 4.238, *p* = .034). Furthermore, fish in the light treatments showed 28% higher C:P ratios than those reared in the shaded treatments (Light: *F*_1,8_ = 6.557, *p* = .034), but there were no differences in N:P ratios in fish reared in the different treatments (*F*_3,8_ = 1.411, *p* = .309).

**Figure 3:**
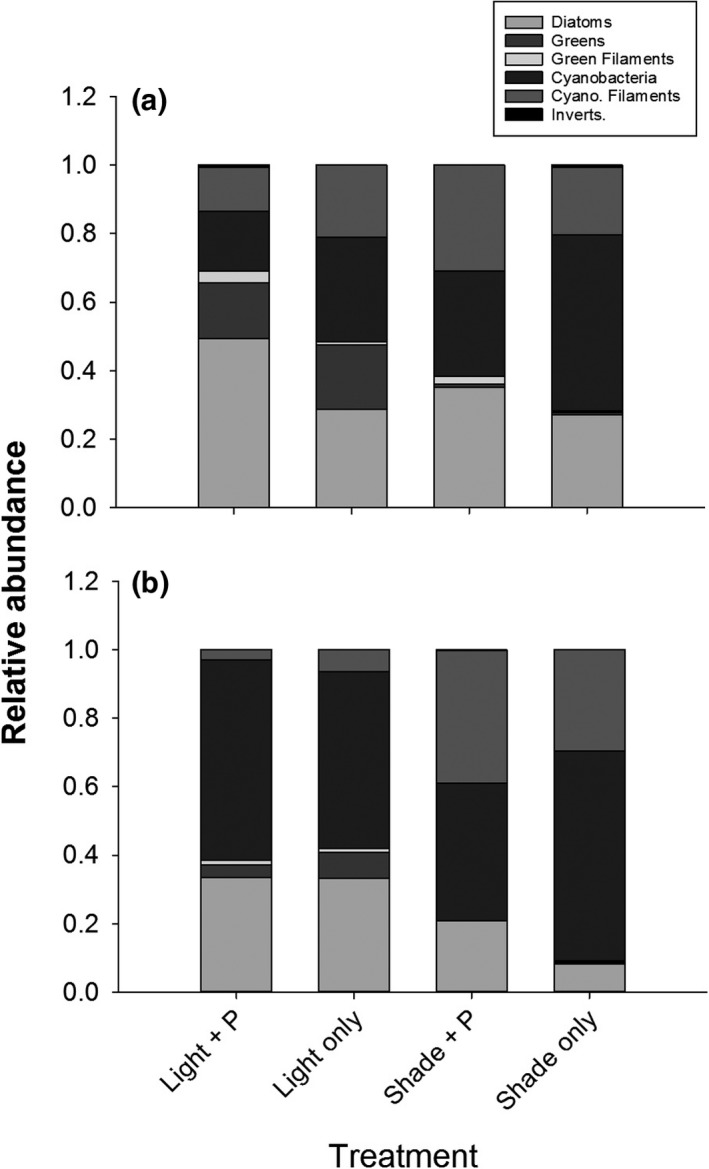
(a) Relative abundance of algal species comprising fish guts reared in various treatments at 3 weeks. Guts are composed of similar proportions of diet items across treatments, and are dominated by diatoms and cyanobacteria. (b) Relative abundance of algal species comprising fish guts reared in various treatments at 6 weeks. Fish guts from light treatments are composed of similar proportions of diet items, and are dominated by cyanobacteria. Those from shaded treatments also contain a high proportion of cyanobacteria, but also have higher proportions of green filamentous algal species than fish guts from the light treatments

**Figure 4:**
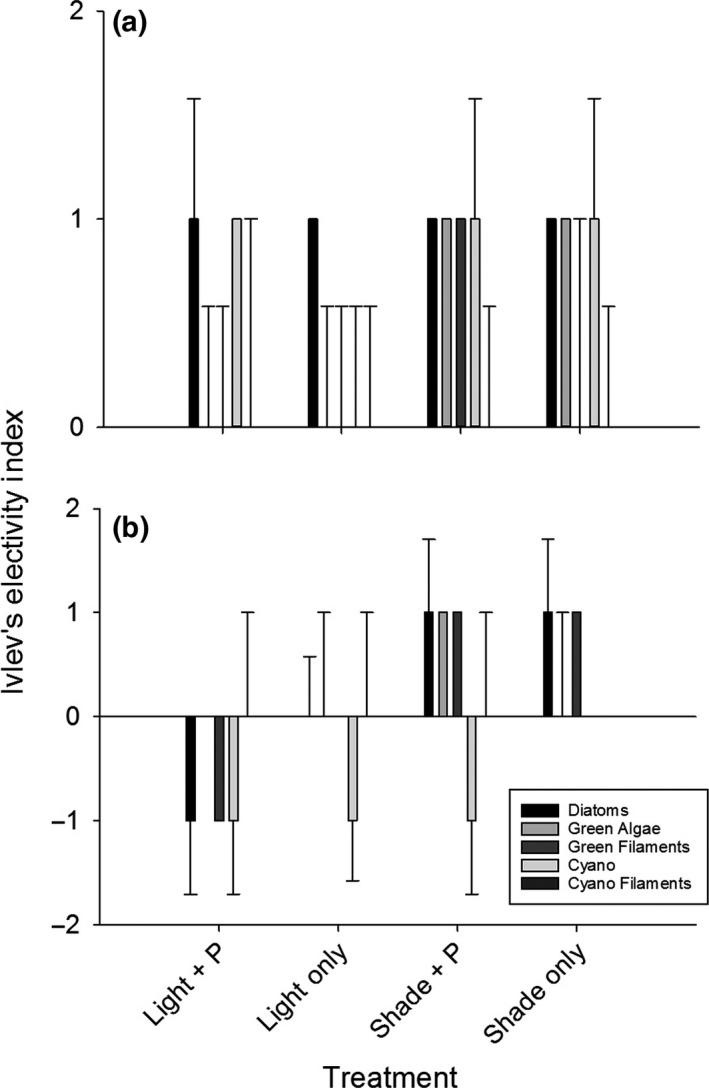
(a) Ivlev's Electivity Index (L_i_) calculated for fish reared in various treatments at 3 weeks. All fish expect those in “Shade + P” cages are actively avoiding filamentous cyanobacteria. (b) Ivlev's Electivity Index (L_i_) calculated for fish reared in various treatments at 6 weeks. Fish reared in “Light + P” cages are avoiding all diet types, whereas, all other fish are only avoiding coccoid cyanobacterial species

The experimental treatments did not affect autotrophic species composition of 3‐week fish guts. The algal composition of 3‐week fish guts (Wilks’ Lambda = 0.253, *F*_18,37_ = 1.297, *p* = .245; Figure 3a) and the relative abundances of edible algae were similar across treatments (*F*_3,8_ = 0.414, *p* = .748). There were some fish with invertebrate parts present in guts at both time periods (<1% of total gut material), but these values were not significantly different across treatments. Although these values were similar, Ivlev's Electivity Index varied for fish eating the different epiphyton types because available food varied among treatments. Indices suggested that fish reared in the light treatments consumed green algal species in proportion to their availability in the environment, whereas those in the shaded treatments actively selected green algae. In addition, fish reared in the “light only” treatment proportionally consumed cyanobacteria as they were available, and fish in the other treatments selectively fed on cyanobacterial species. Fish in all treatments selectively fed on diatoms and consumed cyanobacterial filaments in proportion to their availability (Figure 4a; Table S4).

The differences in relative abundance of fatty acids in fish tissues were subtle at 3 weeks. There were no differences in relative fatty acid content of fish tissues across treatments (*F*_3,8_ = 1.362, *p* = .322), or in the relative abundance of algal and bacterial‐derived fatty acids in the fish tissues across experimental treatments (Wilks’ Lambda = 0.728, *F*_6,14_ = 0.840, *p* = .533).

The relative amounts of PUFAs and SAFAs in fish tissues were marginally different (Wilks’ Lambda = 0.102, *F*_9,15_ = 2.549, *p* = .054). The shaded treatments revealed a 10% increase in PUFAs, whereas “light only” fish had 36% lower SAFA abundance in their tissues. Despite these differences, the SAFA + MUFA: PUFA ratios were the same for fish tissues at 3 weeks (*F*_3,8_ = 2.658, *p* = .120). There were no differences in essential fatty acids (EPA, DHA, ARA) in fish tissues (Wilks’ Lambda = 0.277, *F*_9,15_ = 1.140, *p* = .396), but initial fish tissues had 91% higher DHA than fish tissues from experimental treatments (*F*_4,10_ = 3.940, *p* = .036). For a summary of results, refer to Table 2.

#### 3.3.2 6 weeks

Similar to 3‐week data, there were differences in Sailfin Molly survival, but not growth rate at 6 weeks. The light cages were still 2°C warmer than the shaded cages (*F*_3,51_ = 4.376, *p* = .007), but all cage temperatures decreased by 2°C in the second half of the experiment. This temperature change did not affect fish growth, as all fish grew at similar rates during time period 3–6 weeks (*F*_3,8_ = 1.877, *p* = .212; Figure 2a) and achieved similar sizes at 6 weeks (*F*_3,8_ = 1.425, *p* = .305). Fish raised in the “shade only” treatment experienced 53% higher survival relative to fish reared in the nutrient addition treatments (Χ^2^ = 15.837, *p* < .0001). Fish reared in the “light only” treatments experienced the lowest survival (Figure 2b).

There were stoichiometric differences in fish tissues at 6 weeks. Similar to 3‐week fish tissues, fish in the “light + P” and “light only” treatments had 32% and 45% higher ratios of C:P than “shade + P” and “shade only” fish, respectively (*F*_4,9_ = 24.22, *p* < .001). Fish raised in the light treatments also had higher tissue N:P ratios, at 27% higher than “shade + P” fish and 18% higher than “shade only” fish (*F*_4,9_ = 8.481, *p* = .006).

The algal composition of 6‐week fish guts was marginally different across treatments. Fish reared in “light + P” and “light only” treatments had higher proportions of diatoms (200% increase) and green algae (900% increase) in their guts (Wilks’ Lambda = 0.179, *F*_18,37_ = 1.774, *p* = .077). These fish reared in light treatments also had 99% lower abundances of both coccoid and filamentous cyanobacteria in their guts than fish from the shaded treatments (Figure 3b). However, the proportion of edible algal species present in the guts was not different across treatments (*F*_3,8_ = 0.810, *p* = .523). There were some fish with invertebrate parts present in guts at both time periods (<1% of total gut material), but these values were not significantly different across treatments. Ivlev's Electivity Index (E_i_) reflected differences in fish guts at 6 weeks. Indices suggested that fish reared in the “light + P” treatment avoided diatoms, consumed green algae in proportion to their availability in the environment, and avoided all other algal types. Those in the “light only” treatments consumed all algae in proportion to their availability, except cyanobacteria. Fish in both shaded treatments selectively consumed diatoms. “Shade + P” also selectively chose green algae and avoided cyanobacteria. But “shade only” fish ate green and cyanobacterial species in proportion to their availability in the environment (Figure 4b; Table S4).

Differences in relative abundance of fatty acids in fish tissues were revealed at 6 weeks. The abundance of fatty acids in fish tissues was influenced by light (Light: *F*_1,8_ = 6.641, *p* = .033), where “light + P” fish were comprised of 3× greater fatty acid abundance than “shade only” fish. But, there were no differences between experimental treatments and fatty acid content of initial fish (*F*_3,8_ = 1.362, *p* = .322), or in the relative abundance of algal and bacterial‐derived fatty acids in the fish tissues across experimental treatments (Wilks’ Lambda = 0.430, *F*_6,14_ = 1.051, *p* = .441). At 6 weeks, fish reared in the “shade only” treatments had 19% lower abundances of MUFAs, whereas initial fish tissues were 76% higher in PUFAs compared to experimental fish (Wilks’ Lambda = 0.009, *F*_12,15_ = 5.575, *p* = .002). Ratios of SAFA + MUFA: PUFAs were the same for experimental fish, but were 124% higher than those of initial fish (*F*_4,9_ = 12.203, *p* = .002). At 6 weeks, “shade only” fish had higher abundances of both DHA (60% increase) and ARA (71% increase) in their tissues relative to fish in other treatments (Wilks’ Lambda = 0.082, *F*_9,15_ = 2.931, *p* = .033). Still, initial fish tissues were 84% higher in DHA compared to the experimental treatments at week 6 (*F*_4,10_ = 13.148, *p* = .001). For a summary of results, refer to Table 3. For detailed periphyton results for both 3‐ and 6‐week time periods, refer to Table S3 located in the supplementary material.

### 3.4 Testing adaptive hypotheses

**Figure 5:**
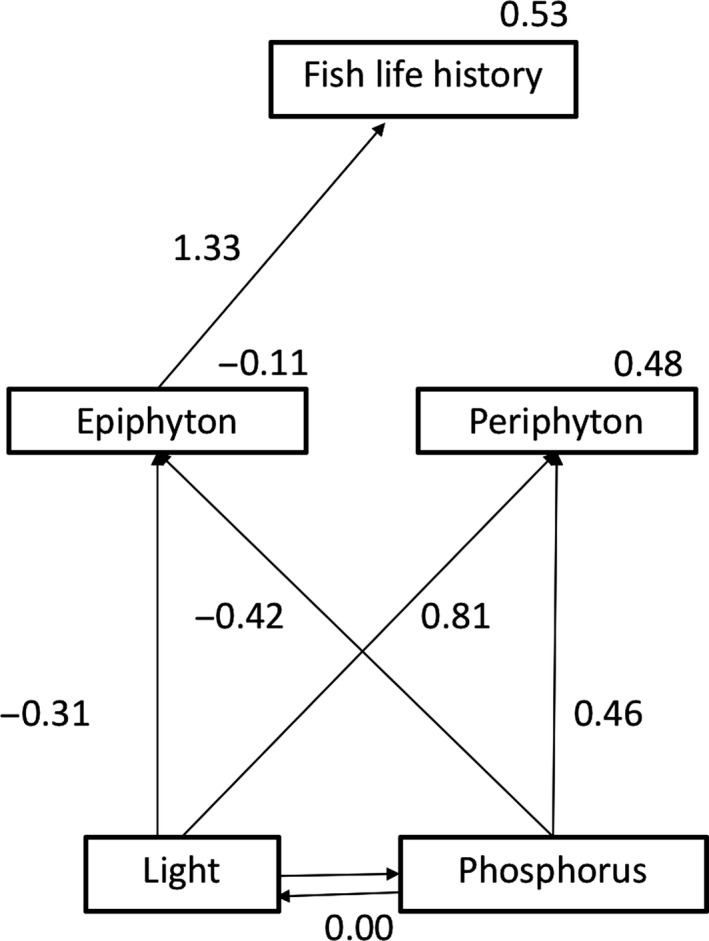
The structural equation model with the best fit showing epiphyton at 3 weeks as the best predictor of fish life history at 3 weeks. Numbers indicate regression coefficients for each path analyzed

Based on ∆AICc values and evidence ratios, SEMs suggested that epiphtyon was the primary food source for Sailfin Mollies in this study (Table 4; Figure 5). In addition, Akaike weights for the alternative models (“epiphyton + periphyton” and “periphyton only”) suggest that the best‐fit model is 3× more likely than the others. Path coefficients for the linkages between periphyton and fish life history were negative in all models, and those between epiphyton and life history were positive in all models, suggesting that epiphyton positively influenced fish life history and periphyton did not. Based on this evidence, we concluded that epiphyton, and not periphyton, was the preferred food source for fish in this study. This information was used to inform the second group of structural equation models that were designed to test the Heterotroph Facilitation and Lipid Allocation hypotheses.

**Table 4:** Comparison of structural equation models used to predict diet type (epiphyton vs. periphyton)

Model	Description	∆AICc	AIC_w_	*w*_min_/*w*_*j*_
1	Epiphyton + Periphyton	2.19	0.20	0.33
2	**Epiphyton**	**0.00**	**0.61**	**1.00**
3	Periphyton	2.38	0.19	0.30

AIC_w_ = Akaike weights, *w*_min_/*w*_*j*_ = Evidence ratios. ∆AICc values ≤2 are highlighted in bold.

**Figure 6:**
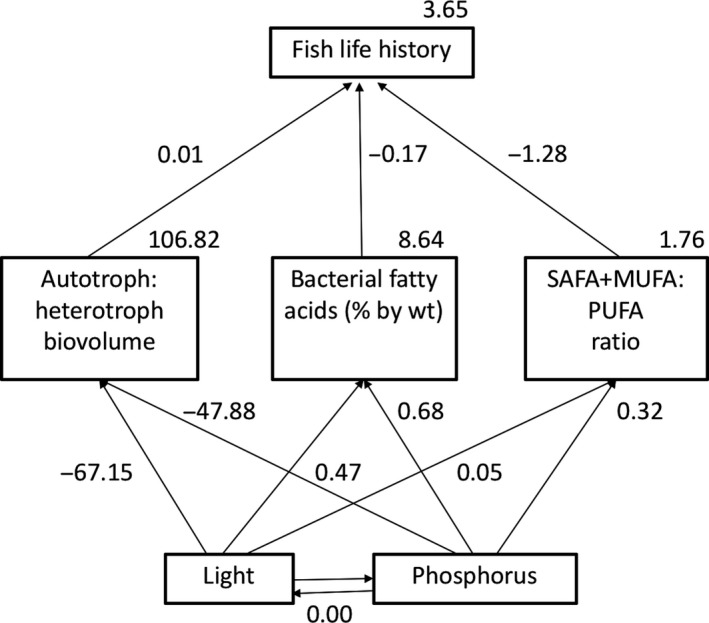
The structural equation model with the best fit showing A:H biovolume, the percentage of bacterial fatty acids and the ratio of SAFA+MUFA:PUFA (FA ratio) at 3 weeks as the best predictor of fish life history at 3 weeks. Numbers indicate regression coefficients for each path analyzed

**Figure 7:**
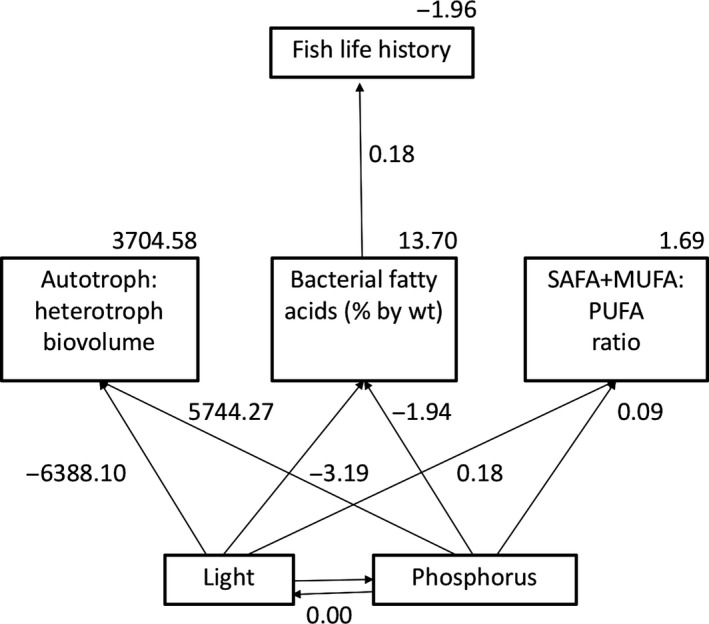
The structural equation model with the best fit showing 6‐week bacterial fatty acid percentage as the best predictor of fish life history at 6 weeks. Numbers indicate regression coefficients for each path analyzed

**Figure 8:**
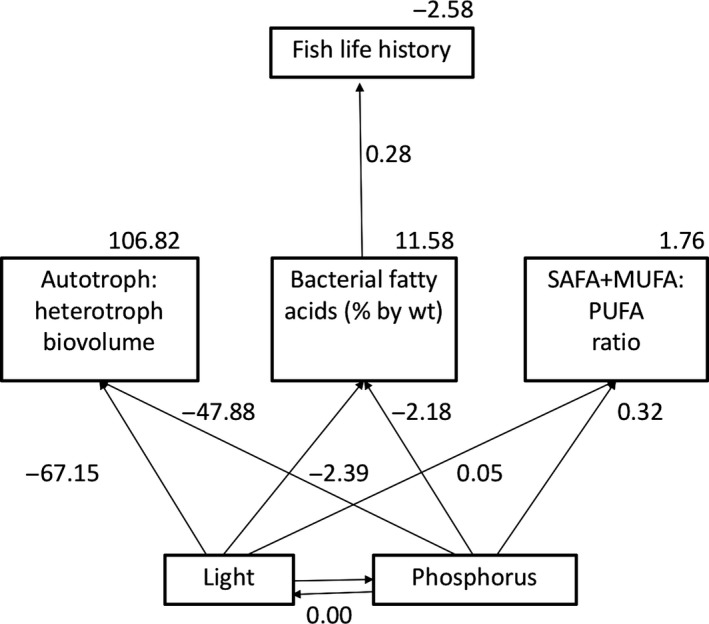
The structural equation model with the best fit showing 3‐week bacterial fatty acids percentage as the best predictor of fish life history at 6 weeks. Numbers indicate regression coefficients for each path analyzed

To test the alternative hypotheses, we varied the paths between diet metrics (A:H biovolume, the percentage of bacterial‐derived fatty acids, SAFA + MUFA:PUFA ratio) and fish life history to produce seven models for each time period, and an additional set of models that linked 3‐week epiphyton characteristics to 6‐week fish. Based on ∆AICc values and evidence ratios, the best fit model suggests that all three diet metrics influence fish life history at 3 weeks. There are several equally supported models (Table 5), but based on the path coefficients, they all suggest that fish life history trait values increase in proportion to A:H biovolume ratio. Path coefficients also show that fish size and survival decrease with increasing bacterial fatty acid percentage and SAFA + MUFA:PUFA ratio at 3 weeks (Figure 6). According to their evidence ratios, these supported models are between 3 and 6× more likely than those with poor fit (∆AICc >2.00). However, at 6 weeks, “A:H + Bac. FA %,” “Bac. FA % + FA ratio” and “Bac. FA %” models were the best supported based on ∆AICc values. Evidence ratios and path coefficients suggest that bacterial fatty acid percentage alone predicts fish life history 3× better than the other supported models, and 3–9× better than the models with no support (Table 5; Figure 7). Models comparing 3‐week diets to diets of 6‐week fish, have similar support as 6‐week models, and also suggest that increased bacterial fatty acid percentage best predicted fish life history (Table 5; Figure 8).

**Table 5:** Comparison of structural equation models used to test “Heterotrophic facilitation” and “Lipid allocation” hypotheses

Model	Description	3 weeks			6 weeks			3→6 weeks		
		∆AICc	AIC_w_	*w*_min_/*w*_*j*_	∆AICc	AIC_w_	*w*_min_/*w*_*j*_	∆AICc	AIC_w_	*w*_min_/*w*_*j*_
1	A:H + Bac. FA + FA ratio	**0.00**	**0.26**	**1.00**	3.95	0.05	0.14	3.28	0.07	0.19
2	A:H + Bac. FA	**0.32**	**0.22**	**0.85**	**1.95**	**0.15**	**0.38**	**1.91**	**0.15**	**0.38**
3	A:H + FA ratio	**0.62**	**0.19**	**0.73**	4.36	0.04	0.11	4.16	0.05	0.12
4	Bac. FA + FA ratio	2.36	0.08	0.31	**2.00**	**0.14**	**0.37**	**1.32**	**0.20**	**0.51**
5	A:H	**1.77**	**0.11**	**0.41**	2.36	0.12	0.31	2.23	0.12	0.33
6	Bac. FA	2.15	0.09	0.34	**0.00**	**0.39**	**1.00**	**0.00**	**0.38**	**1.00**
7	FA ratio	3.73	0.04	0.15	2.49	0.11	0.29	5.15	0.03	0.08

A:H = A:H biovolume, Bac. FA = percentage of bacterial fatty acids, FA ratio = SAFA + MUFA:PUFA ratio. AIC_w_ = Akaike weights, *w*_min_/*w*_*j*_ = Evidence ratios. ∆AICc values ≤2 are highlighted in bold.

## 4 DISCUSSION

We found evidence that detritivory facilitates herbivory, supporting the suggestion that “true” herbivory is rare in nature (White, 1985). Our study indicated that herbivorous Sailfin Mollies benefit from a diet supplemented with heterotrophic microbes, consistent with the Heterotroph Facilitation hypothesis. In our experiment, increased algal biovolume, increased proportion of monounsaturated fatty acids, and decreased percentage of bacterial fatty acids in the diet best predicted early Sailfin Molly life history (6–9 weeks of age). However, later in development (9–12 weeks of age), cages with high heterotroph fatty acid production yielded the highest juvenile survival. These results indicate that prior to maturation, Sailfin Mollies benefit from a mixed diet of autotrophic and heterotrophic food sources. The Lipid Allocation hypothesis focuses on algal‐derived lipids as the main driver of herbivore success and was therefore not supported in this study. Rather, we show that heterotrophs supplement algal diets, and the quality (e.g., fatty acid abundance) of these microbes strongly influences herbivore life history by increasing survival by up to 53%. However, because Sailfin Mollies did not reach sexual maturity at the end of this experiment, we are unable to determine any potential trade‐offs between survival and reproductive output, or if heterotrophic bacteria are important in the reproductive phase. Furthermore, our findings do not explain why herbivory exists as an alternative to a carnivorous diet, although we do provide a justification for how herbivory is sustained in a natural setting. Finally, our findings confirm that “herbivory” in aquatic systems may routinely include detritivory and that “green” food webs may be less common than thought Change to Moore et al. 2004:

Although some authors have examined the influence of dietary heterotrophs on herbivore life history (e.g., Belicka et al., 1993; Bowen, 1966; Smoot & Findlay, 2010), it is not typically recognized as a fundamental part of the herbivorous diet (White, 1985). Many studies have assessed diet quality effects on life history using stoichiometry, polyunsaturated fatty acids, or indices like algal edibility, but, these diet measures were not retained in the model that best fit our data. The ecological stoichiometry literature assumes that diets with lower C:P ratios are the highest quality for consumers, and consumer tissues will reflect these diets by having high C:P levels (Sterner & Elser, 2002). This was not the case in our study as fish with the highest survival (“shade only”) were consuming epiphyton with high C:P ratios and had tissues with low C:P ratios, although P did not appear to be limiting in the diet of fish in our field cages. This finding was not surprising because animals catabolize and metabolize molecules, not individual elements (Raubenheimer, Simpson, & Mayntz, 2009; Sperfeld et al., 2017). The nutritional ecology literature suggests that food items with high PUFA content are of higher quality (e.g., Müller‐Navarra et al., 2004; Persson & Vrede, 2006), but we show that the highest surviving fish (“shade only”) consumed epiphyton with low SAFA + MUFA: PUFA ratios, similar to “light only” fish who showed relatively low survival. Edibility indices have also been used as a simple measure of food quality (e.g., Geddes & Trexler, 2003; Trexler et al., 2015), where higher proportion of green algae and diatoms relative to cyanobacteria indicates a higher quality food source (Lamberti, 1996; Stienman, 1996; Sullivan & Currin, 2000). In our study, fish with high survival (“shade only”) were in cages with epiphyton with relatively high abundances of both filamentous and coccoid cyanobacteria. However, Ivlev's Electivity index showed that fish were feeding selectively on higher quality food items when they were not abundant in the environment. This suggests that estimations of food quality that are derived from edibility indices are compromised by feeding strategies and are thus not reliable indicators of food quality. If this study had been conducted with a higher density of fish, increasing competition and precluding selective feeding, our results may have differed. The density used was reflective of ambient densities in the study area.

While our study did not find support for the Lipid Allocation hypothesis, algal‐derived fatty acids are important to herbivores. Fatty acids originating from primary producers fuel growth, survival, and reproduction of herbivores, but our results emphasize that life history characteristics are optimized when these diets are supplemented with heterotrophs (e.g., Martin‐Creuzburg et al., 2005, 2011). We found that diets with high levels of both bacterial‐derived fatty acids and PUFAs (e.g., “shade + P” epiphyton) were suboptimal for herbivore survival. Similarly, diets with intermediate levels of PUFAs, and decreased bacterial‐derived fatty acids (e.g., “light only”), or diets with decreased levels of both fatty acid types (e.g., “light + P”) are not ideal for herbivores. Diets with intermediate levels of PUFAs (e.g., “shade only”) were the best available diets in this study, providing evidence that detritivory represents an important part of the herbivorous diet as predicted by the Heterotroph Facilitation hypothesis.

We began this research to explore the conditions that would favor the evolution of an herbivorous diet from a carnivorous or omnivorous one. This study suggests that including heterotrophic microbes in the diet can compensate for the generally poor quality of aquatic plant foods. However, this study does not address how other nutritional components (e.g., macronutrients, algal starch, etc.) may have changed in response to our experimental manipulations, or their interactive effects on herbivore life history. Furthermore, we are unable to conclude why carnivory would be largely abandoned in herbivore‐detritivores like Sailfin Mollies. Other adaptive hypotheses outlined by Sanchez and Trexler (2016) may fill this gap. For example, ancestral herbivores may have invaded habitats with few predators and animal prey, but high in microbial and autotrophic biofilms. Because the mechanisms supporting the evolution of herbivory remain unknown, we hope this study is a step in establishing a research framework that will allow us to more fully understand herbivory from an adaptation perspective.

## CONFLICT OF INTEREST

None declared.

## AUTHOR CONTRIBUTION

J.L.S. and J.C.T. conceived the ideas presented in this manuscript. J.L.S. collected and analyzed data, and led the writing of the manuscript. J.C.T. assisted with experimental design, statistical analyses and contributed to editing the manuscript.

## DATA ACCESSIBILITY

Data from: When is an herbivore not an herbivore? Detritivory facilitates herbivory in a freshwater system. Dryad Digital Repository. https://doi.org/10.5061/dryad.jd6875k
