# Supplementary material for: When is an herbivore not an herbivore? Detritivory facilitates herbivory in a freshwater system
Source: Ecol Evol. 2018 May 7;8(12):5977–91. doi: 10.1002/ece3.4133 (PMC6024117; doi:10.1002/ece3.4133)
Supplement: Supplementary file 1 [file ECE3-8-5977-s001.docx]

**Electronic Supplementary Material:**

**When is an herbivore not an herbivore? Detritivory facilitates herbivory in a freshwater system**

Jessica L Sanchez* and Joel C Trexler

Department of Biological Sciences, Florida International University, Miami, USA

*Corresponding author: jsanc318@fiu.edu

**Figure S1.** Field experimental set-up. Boxes represent 1m^2^ mesh cages (shaded and open) randomly distributed across a 980 m^2^ plot located in an open Everglades slough (25°49’41.23”N, 80°37’53.41”W). Not drawn to scale.


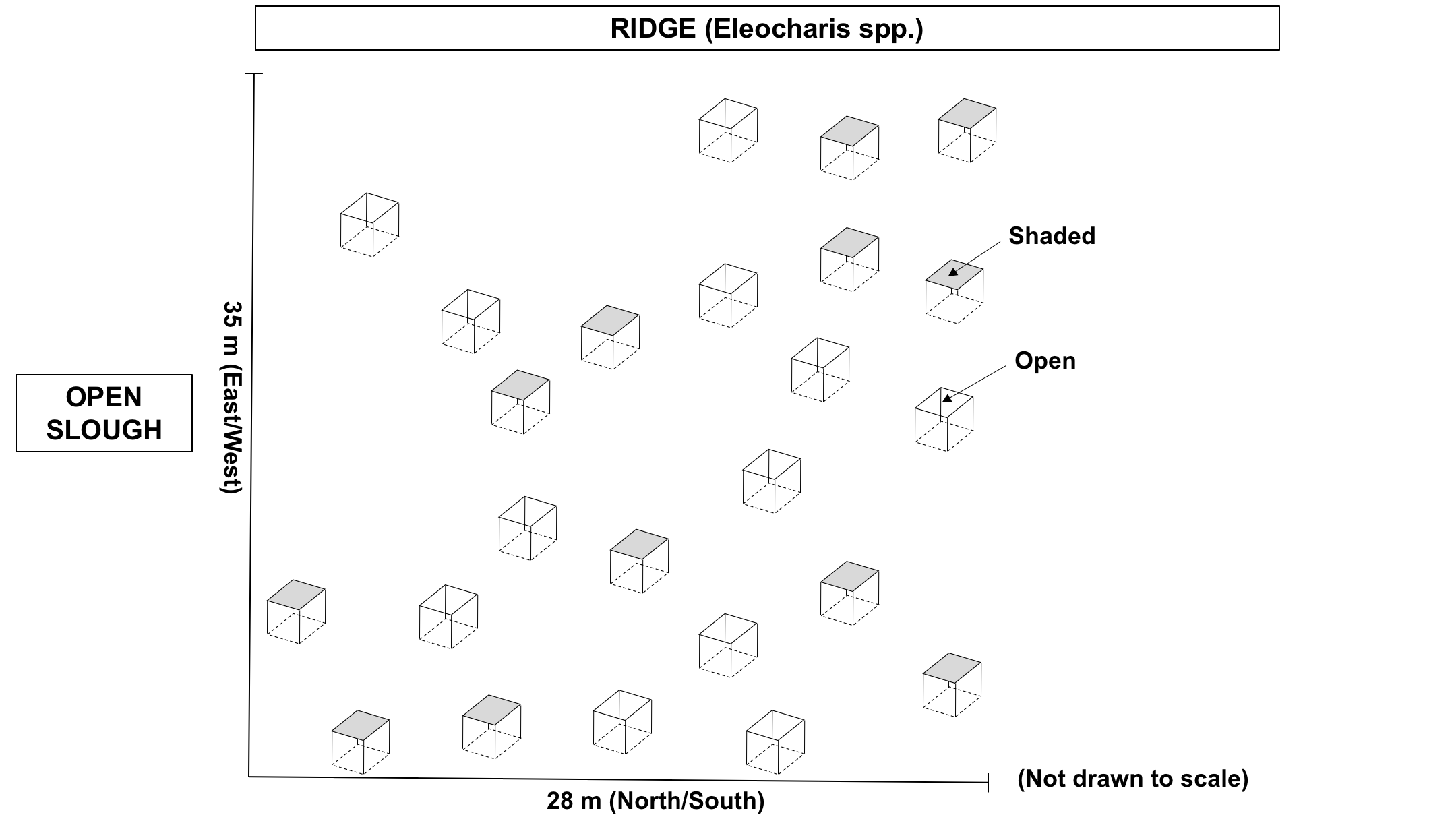


**Figure S2.** (a) Photo showing mesh cages in the field. (b) Photo showing cages wrapped with 3mm clear plastic following nutrient dosing. Phosphorus (Na_2_HPO_4_) was added once per week and the cages remained wrapped for 24 hours to avoid seepage to cages without nutrient addition.


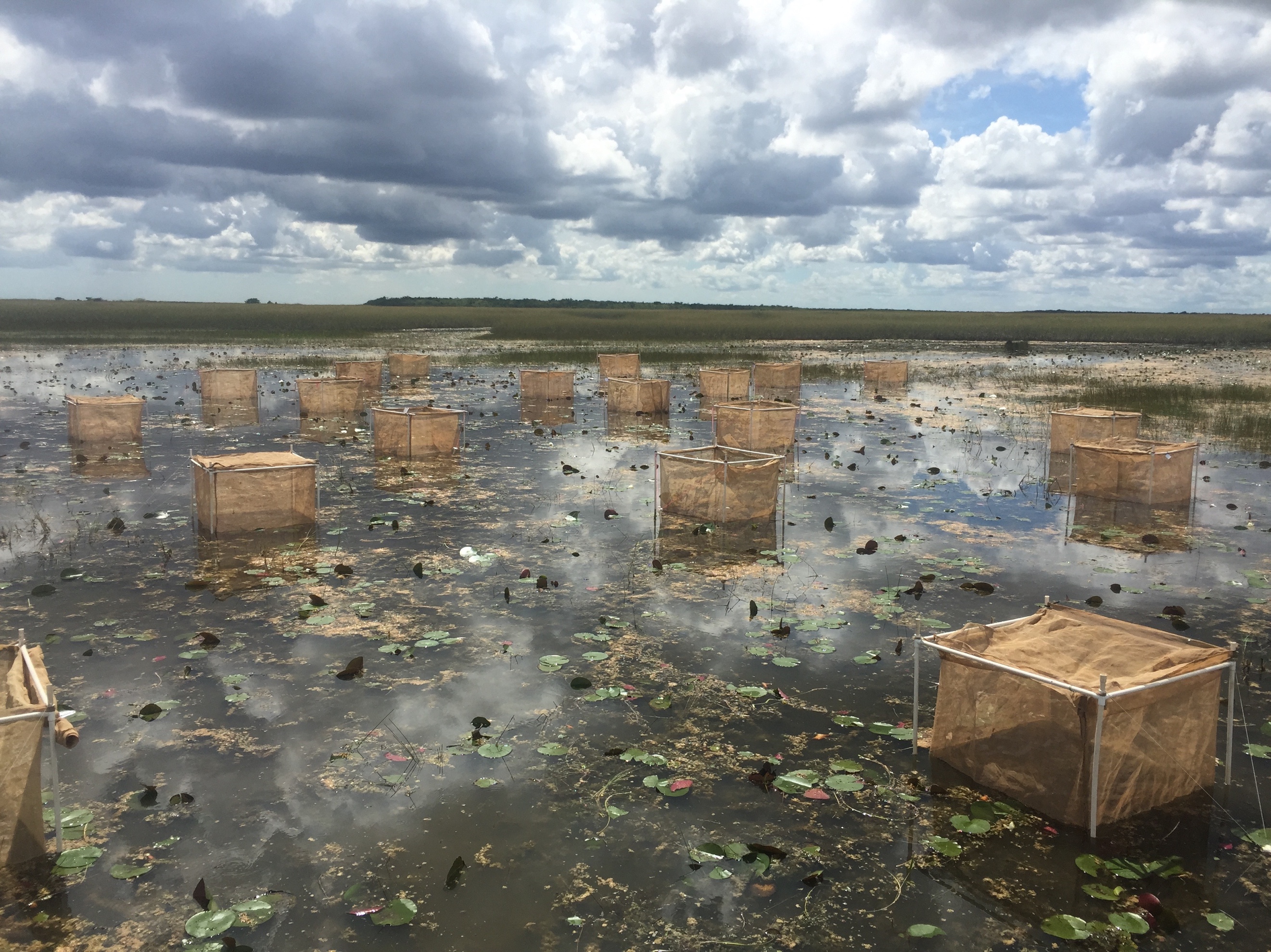


**(a)**


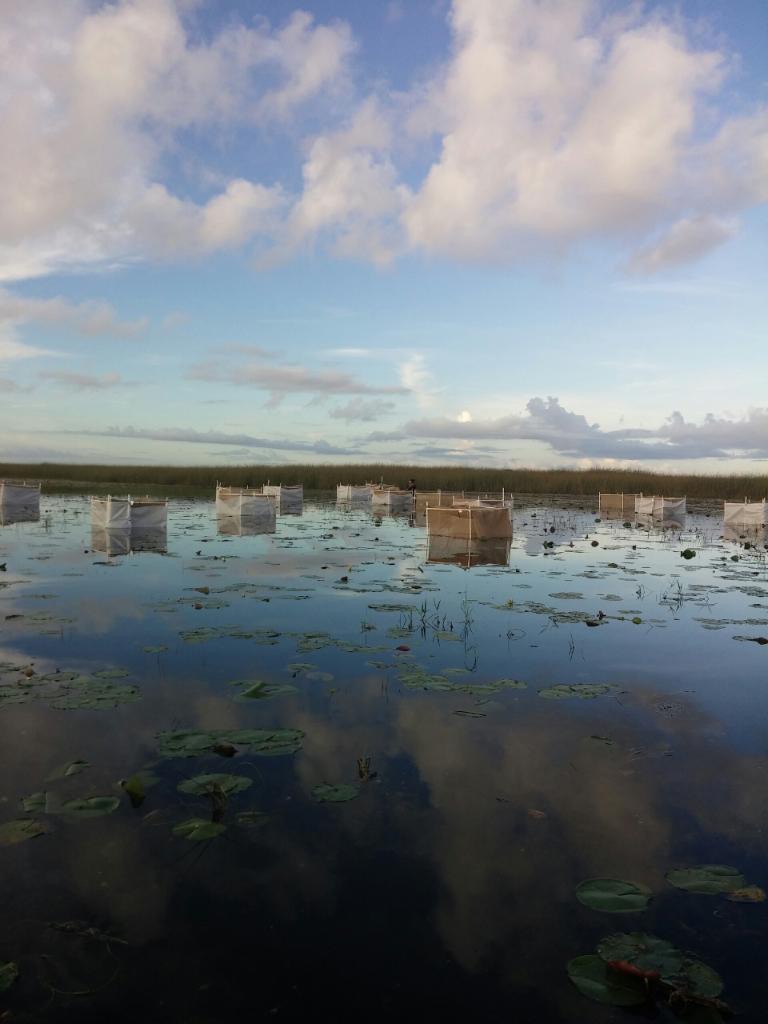


**(b)**

| **Treatment** | **C:P** | **N:P** | **A:H biovolume** | **Bac. FA (% by weight)** | **Algal FA (% by weight)** | **PUFA (% by weight)** | **SAFA (% by weight)** | **MUFA (% by weight)** | **EPA:DHA** | **ARA (% by weight)** | **Proportion of edible algal spp.** |
| --- | --- | --- | --- | --- | --- | --- | --- | --- | --- | --- | --- |
| **Light+ P** |  |  |  |  |  |  |  |  |  |  |  |
| 3 weeks  6 weeks | 2058.20 + 301.82  1460.23 + 424.87 | 162.43 + 30.37  105.81 + 29.06 | 12.44 + 5.13  147.83 + 2.38 | 10.26 + 3.54  8.81 + 1.05 | 27.23 + 6.63  33.09 + 0.05 | 9.39 + 0.46  12.71 + 6.35 | 57.33 + 0.14  55.64 + 9.99 | 33.08 + 0.45  31.65 + 3.65 | 3.73 + 4.35  5.76 + 6.86 | 1.60 + 0.59  2.40 + 0.18 | 0.82 + 0.09  0.54 + 0.33 |
| **Light only** |  |  |  |  |  |  |  |  |  |  |  |
| 3 weeks  6 weeks | 1995.80 + 87.11  2119.64 + 411.12 | 147.95 + 6.29  149.72 + 26.21 | 19.01 + 9.02  229.39 + 73.52 | 8.63 + 0.41  10.27 + 2.20 | 29.85 + 1.29  29.63 + 5.09 | 15.89 + 3.21  14.43 + 2.43 | 53.69 + 0.65  54.46 + 4.11 | 30.42 + 2.58  31.12 + 2.14 | 3.95 + 0.87  4.44 + 23.44 | 1.93 + 0.16  2.09 + 0.33 | 0.97 + 0.06  0.95 + 0.07 |
| **Shade + P** |  |  |  |  |  |  |  |  |  |  |  |
| 3 weeks  6 weeks | 1999.73 + 272.93  1984 + 87.55 | 149.76 + 20.31  148.89 + 15.22 | 38.29 + 23.56  12361.76 + 9805.99 | 8.84 + 2.04  11.52 + 2.44 | 32.50 + 0.49  30.90 + 4.28 | 12.50 + 1.08  15.49 + 0.75 | 52.81 + 0.97  52.06 + 3.03 | 34.69 + 0.65  32.44 + 2.65 | 4.89 + 2.55  4.36 + 2.44 | 1.65 + 0.33  1.97 + 0.36 | 0.53 + 0.30  0.78 + 0.03 |
| **Shade only** |  |  |  |  |  |  |  |  |  |  |  |
| 3 weeks  6 weeks | 2058.88 + 108.58  2129.14 + 89.84 | 154.97 + 2.11  145.91 + 15.42 | 127.47 + 73.98  791.66 + 279.70 | 9.11 + 2.03  13.94 + 2.20 | 32.42 + 1.77  27.19 + 1.41 | 13.11 + 0.43  14.59 + 1.35 | 52.73 + 1.40  54.05 + 0.95 | 34.15 + 1.81  31.35 + 0.73 | 4.55 + 1.09  4.10 + 7.54 | 1.68 + 0.11  1.84 + 0.09 | 0.49 + 0.13  0.46 + 0.09 |

**Table S1.** Average values + 1 SD for all measured epiphyton variables by treatment

**Table S2.** Average values + 1 SD for all measured periphyton variables by treatment. NA= variables that could not be measured for that treatment

*values were both zero and thus, the ratio was unable to be calculated

| **Treatment** | **C:P** | **N:P** | **A:H biovolume** | **Bac. FA (% by weight)** | **Algal FA (% by weight)** | **PUFA (% by weight)** | **SAFA (% by weight)** | **MUFA (% by weight)** | **EPA:DHA** | **ARA (% by weight)** | **Proportion of edible algal spp.** |
| --- | --- | --- | --- | --- | --- | --- | --- | --- | --- | --- | --- |
| **Ambient** | 5298.94 | 172.54 | 8.34 | 13.43 | 18.30 | 12.38 | 60.28 | 27.34 | NA* | 1.16 | 0.12 |
| **Light+ P** |  |  |  |  |  |  |  |  |  |  |  |
| 3 weeks  6 weeks | 4585.99 + 759.79  3343.42 + 850.53 | 191.80 + 30.90  165.33 + 29.58 | 17.35 + 7.31  5.34 + 3.53 | 10.34 + 2.64  14.95 + 2.83 | 22.29 + 2.24  16.70 + 1.40 | 16.04 + 3.20  16.27 + 3.47 | 51.77 + 2.62  53.05 + 3.81 | 32.18 + 1.85  30.68 + 3.16 | 65.33 + 33.62  57.53 + 9.35 | 1.72 + 0.43  1.28 + 0.17 | 0.74 + 0.23  0.30 + 0.09 |
| **Light only** |  |  |  |  |  |  |  |  |  |  |  |
| 3 weeks  6 weeks | 4349.86 + 829.44  3513.10 + 540.84 | 179.25 + 23.88  187.49 + 43.48 | 21.65 + 23.33  133.19 + 93.37 | 9.66 + 0.88  15.98 + 1.44 | 21.86 + 2.47  17.05 + 3.12 | 18.63 + 1.11  14.01 + 1.82 | 30.03 + 0.93  56.19 + 5.33 | 30.03 + 0.93  29.80 + 3.53 | 65.92 + 24.11  20.27 + 85.12 | 1.60 + 0.39  1.29 + 0.45 | 0.57 + 0.10  0.27 + 0.09 |
| **Shade + P** |  |  |  |  |  |  |  |  |  |  |  |
| 3 weeks  6 weeks | 4027.97 + 358.06  3020.67 + 343.04 | 191.29 + 25.26  162.36 + 27.84 | 21.39 + 8.11  18.27 + 14.07 | 7.97 + 0.80  15.13 + 1.00 | 23.01 + 3.87  16.14 + 1.57 | 20.05 + 2.90  13.11 + 0.75 | 51.22 + 0.97  57.66 + 4.37 | 28.74 + 3.85  29.23 + 3.64 | 59.60 + 28.98  35.96 + 16.01 | 1.46 + 0.52  1.11 + 0.11 | 0.35 + 0.13  0.19 + 0.13 |
| **Shade only** |  |  |  |  |  |  |  |  |  |  |  |
| 3 weeks  6 weeks | 3995.32 + 570.69  3019.93 + 600.33 | 194.55 + 15.17  161.18 + 25.77 | 4.39 + 2.32  20.74 + 11.02 | 8.62 + 3.14  15.63 + 1.40 | 23.68 + 2.47  17.25 + 1.62 | 19.26 + 2.81  14.43 + 1.74 | 51.23 + 0.78  54.89 + 1.11 | 29.50 + 3.15  30.68 + 1.46 | 52.02 + 46.37  23.85 + 20.02 | 1.36 + 0.18  1.10 + 0.15 | 0.52 + 0.16  0.24 + 0.05 |

**Table S3.** Average values + 1 SD for all measured fish variables by treatment. NA= variables that could not be measured for that treatment

| **Treatment** | **Survival score (*p’*)** | **Size (mm)** | **C:P** | **N:P** | **Bac. FA (% by weight)** | **Algal FA (% by weight)** | **PUFA (% by weight)** | **SAFA (% by weight)** | **MUFA (% by weight)** | **EPA:DHA** | **ARA (% by weight)** |
| --- | --- | --- | --- | --- | --- | --- | --- | --- | --- | --- | --- |
| **Initial** | NA | NA | 54.37 | 10.50 | 5.09 | 22.30 | 37.13 | 31.77 | 31.09 | 0.09 | 3.43 |
| **Light+ P** |  |  |  |  |  |  |  |  |  |  |  |
| 3 weeks  6 weeks | 0.78 + 0.10  0.95 + 0.09 | 18.30 + 0.53  22.63 + 2.34 | 108.79 + 18.57  95.33 + 5.01 | 20.54 + 4.75  17.71 + 1.93 | 10.90 + 1.76  11.14 + 3.15 | 31.83 + 1.65  35.30 + 3.04 | 22.88 + 4.25  19.39 + 1.23 | 45.59 + 0.99  43.60 + 1.96 | 31.54 + 3.64  37.01 + 1.51 | 0.18 + .05  0.27 + 0.18 | 5.17 + 1.56  3.49 + 0.26 |
| **Light only** |  |  |  |  |  |  |  |  |  |  |  |
| 3 weeks  6 weeks | 0.83 + 0.00  0.90 + 0.001 | 20.03 + 1.40  22.1 + 1.74 | 110.62 + 7.10  93.51 + 5.58 | 21.24 + 0.74  17.71 + 1.93 | 11.50 + 1.19  12.04 + 1.87 | 30.29 + 0.91  31.73 + 2.21 | 21.93 + 1.10  20.05 + 1.70 | 33.21 + 1.32  44.72 + 0.70 | 33.21 + 1.32  35.23 + 2.41 | 0.22 + 0.69  0.27 + 0.05 | 4.14 + 0.38  3.75 + 0.49 |
| **Shade + P** |  |  |  |  |  |  |  |  |  |  |  |
| 3 weeks  6 weeks | 1.04 + 0.06  0.91 + 0.02 | 18.80 + 1.61  21.83 + 0.81 | 85.78 + 17.88  70.63 + 7.04 | 17.11 + 3.44  13.68 + 0.59 | 10.35 + 1.59  12.32 + 1.39 | 28.42 + 3.26  31.20 + 11.29 | 24.30 + 1.61  21.66 + 1.08 | 45.46 + 0.37  44.12 + 1.76 | 30.24 + 1.39  34.23 + 0.68 | 0.15 + 0.16  0.24 + 0.10 | 5.28 + 0.69  4.02 + 0.02 |
| **Shade only** |  |  |  |  |  |  |  |  |  |  |  |
| 3 weeks  6 weeks | 1.12 + 0.01  1.39 + 0.53 | 19.10 + 1.57  20.23 + 0.70 | 85.67 + 18.37  64.54 + 5.39 | 16.79 + 3.17  14.01 + 0.87 | 10.48 + 0.73  11.15 + 0.24 | 31.40 + 1.58  30.82 + 0.17 | 27.55 + 1.50  24.66 + 5.57 | 45.31 + 0.61  45.84 + 1.55 | 27.14 + 1.04  28.61 + 2.75 | 0.12 + 0.06  0.17 + 0.11 | 6.31 + 0.37  5.99 + 1.56 |

**Table S4.** Average values + 1 SD for Ivlev’s Electivity Index by treatment. NA= variables that could not be measured for that treatment

| **Treatment** | **Diatoms** | **Green Algae** | **Green Filaments** | **Cyano.** | **Cyano. Filaments** |
| --- | --- | --- | --- | --- | --- |
| **Light+ P** |  |  |  |  |  |
| 3 weeks  6 weeks | 1.00 + 0.58  -1.00 + 0.71 | 0.00 + 0.58  0.00 + 0.00 | 0.00 + 0.58  -1.00 + 0.00 | 1.00 + 0.00  -1.00 + 0.71 | 0.00 + 1.00  0.00 + 1.00 |
| **Light only** |  |  |  |  |  |
| 3 weeks  6 weeks | 1.00 + 0.00  0.00 + 0.58 | 0.00 + 0.58  0.00 + 1.00 | 0.00 + 0.58  0.00 + 0.00 | 0.00 + 0.58  -1.00 + 0.58 | 0.00 + 0.58  0.00 + 1.00 |
| **Shade + P** |  |  |  |  |  |
| 3 weeks  6 weeks | 1.00 + 0.00  1.00 + 0.71 | 1.00 + 0.00  1.00 + 0.00 | 1.00 + 0.00  1.00 + 0.00 | 1.00 + 0.58  -1.00 + 0.71 | 0.00 + 0.58  0.00 + 1.00 |
| **Shade only** |  |  |  |  |  |
| 3 weeks  6 weeks | 1.00 + 0.00  1.00 + 0.71 | 1.00 + 0.00  0.00 + 1.00 | 0.00 + 1.00  1.00 + 0.00 | 1.00 + 0.58  0.00 + 0.00 | 0.00+ 0.58  0.00 + 0.00 |
